# Comprehensive Transcriptomic and m6A Epitranscriptomic Analysis Reveals Colchicine-Induced Kidney Toxicity via DNA Damage and Autophagy in HK2 Cells

**DOI:** 10.3390/toxins17080408

**Published:** 2025-08-14

**Authors:** Kun Tian, Jiaxin Wen, Dongcheng Zhang, Jiaxuan Lin, Lixiang Weng, Lele Yang, Wei Zhao, Chutao Li, An Zhu

**Affiliations:** 1 Key Laboratory of Gastrointestinal Cancer (Fujian Medical University), Ministry of Education, Fuzhou 350108, China; tiankun@stu.fjmu.edu.cn (K.T.); jiaxin-wen@fjmu.edu.cn (J.W.); zhangdongcheng@stu.fjmu.edu.cn (D.Z.); 3220119057@stu.fjmu.edu.cn (J.L.); wlx@fjmu.edu.cn (L.W.); yanglele@fjmu.edu.cn (L.Y.); zhaowei@fjmu.edu.cn (W.Z.); lichutao@fjmu.edu.cn (C.L.); 2 Key Laboratory of Environment and Health, School of Public Health, Fujian Medical University, Fuzhou 350108, China

**Keywords:** colchicine, m6A modification, DNA damage, autophagy

## Abstract

Colchicine is commonly prescribed for inflammation and gout, but its nephrotoxicity and underlying mechanisms remain incompletely understood. The objective of this research was to clarify the association between m6A methylation modifications and nephrotoxicity caused by colchicine. A significant decrease in HK2 cell viability was observed following colchicine treatment, and mRNA sequencing (mRNA-seq) revealed the differential expression of genes associated with DNA damage and autophagy. Further methylated RNA immunoprecipitation sequencing (MeRIP-seq) analysis revealed an association between N6-methyladenosine (m6A) modifications and the expression of genes involved in DNA damage and autophagy after colchicine exposure. Molecular docking and a molecular dynamics (MD) analysis identified ZC3H13 as a potential regulator of colchicine-induced cytotoxicity in HK2. Experimental validation confirmed that colchicine induces DNA damage and autophagy in HK2 cells, with ZC3H13 playing a significant role in these processes. In conclusion, the findings suggested that colchicine-induced damage in HK2 cells is associated with changes in m6A methylation levels in target genes and the altered expression of m6A regulator.

## 1. Introduction

Derived from the lily family plants *Colchicum autumnale*, colchicine is a bioactive alkaloid that has been used for pain and inflammation relief since its earliest documentation in the Egyptian Ebers Papyrus circa 1550 BCE [1,2]. By suppressing leukocyte activity, reducing collagen synthesis, enhancing collagenase activity, and inducing mitotic arrest alongside concomitant inhibition of DNA synthesis, colchicine has collectively established itself as the cornerstone anti-inflammatory therapy for gout and familial Mediterranean fever [3,4].

Despite its clinical utility, overdose with colchicine triggers severe multiorgan toxicity. Its hepatic metabolism predominates through cytochrome P450 3A4 and P-glycoprotein-mediated biliary excretion, with 10–12% renal clearance, contraindicating hepatorenal impairment [5]. Nephrotoxicity manifests prominently as azotemia, proteinuria, and hematuria, progressing to oliguria or acute renal failure requiring dialysis [6]. Mechanistically, renal injury is correlated with direct toxicity, exemplified by a 5-fold surge in the renal isoform of serum alkaline phosphatase within 48 h following the ingestion of 12.5 mg, in parallel with elevated urea and creatinine levels. Recent studies have revealed that colchicine at low doses attenuated doxorubicin-induced cardiotoxicity through microtubule-regulated autolysosomal degradation and improved renal outcomes in chronic kidney disease by modulating innate immunity [7].

Recent research has shown that the toxicological effects of traditional herbal medicines are linked to m6A, the most common epitranscriptomic modification in eukaryotic mRNAs [8,9,10]. The dynamic regulation of m6A was mediated by methyltransferases such as the METTL3/METTL14 complex and demethylases including FTO and ALKBH5 [11]. m6A broadly participated in cellular differentiation, stress responses, and disease pathogenesis by modulating RNA splicing, stability, transport, and translation. Studies on m6A have revealed its critical roles in DNA damage repair and regulation of autophagy, with potential cross-talk with the biological effects of colchicine. Colchicine induced DNA damage, exhibited m6A regulatory capacity and enhanced global mRNA stability by inhibiting m6A modifications mediated by METTL14 and YTHDC1 [12,13]. Concurrently, m6A critically regulated the repair of DNA damage. For example, METTL3-dependent m6A modifications facilitated the recruitment of homologous recombination repair genes BRCA1 and RAD51, promoting genomic restoration [14]. Autophagy, a key mechanism for clearing damaged organelles, was precisely controlled by m6A through core genes such as ATG5 and LC3. The FTO-mediated stabilization of ATG5 and ATG7 mRNAs increased autophagosome formation, consequently facilitating autophagy [15]. By dynamically coordinating DNA repair and autophagy-related gene expression, m6A has emerged as a central node linking colchicine-induced genomic instability to cellular stress adaptation. This study systematically investigated this regulatory network, exploring colchicine-triggered DNA damage and activation of autophagy. The findings provided a theoretical foundation for optimizing colchicine applications.

## 2. Results

### 2.1. Structural and Purity Determination and Potential Targets of Colchicine

**Figure 1:**
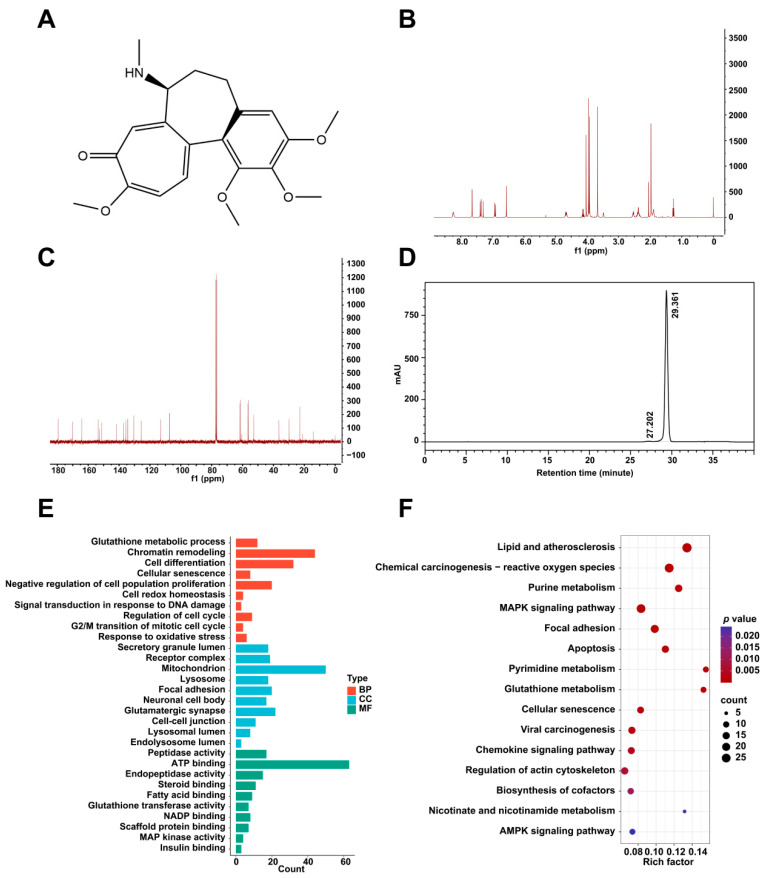
(**A**) The chemical structure of colchicine. (**B**) ^1^H NMR and (**C**) ^13^C NMR spectra of colchicine recorded at 400 MHz. (**D**) HPLC analysis of colchicine purity. (**E**) GO and (**F**) KEGG pathway enrichment analyses of potential target proteins of colchicine derived from the PharmMapper database.

The chemical structure of colchicine was determined via nuclear magnetic resonance (NMR) spectroscopy (Figure 1A). The ^1^H and ^13^C NMR spectra (Figure 1B,C) exhibited characteristic signals consistent with the molecular formula C_22_H_25_NO_6_. A purity analysis via high-performance liquid chromatography (HPLC) revealed a single major peak with a retention time of 29.36 min (Figure 1D). Its purity was determined to be 99.35% via the peak area normalization method.

Using the PharmMapper server (version 2017), 298 potential target proteins interacting with colchicine were identified. Gene Ontology (GO) and Kyoto Encyclopedia of Genes and Genomes (KEGG) pathway enrichment analyses of the predicted targets were conducted using the DAVID bioinformatics database. The GO enrichment analysis included chromatin remodeling, signal transduction in response to DNA damage, focal adhesion, lysosome, and regulation of cell cycle (Figure 1E). The KEGG pathway enrichment results comprised cellular senescence, glutathione metabolism, and AMPK signaling pathway (Figure 1F).

### 2.2. The Cell Viability Effect of Colchicine on HK2 Cells

**Figure 2:**
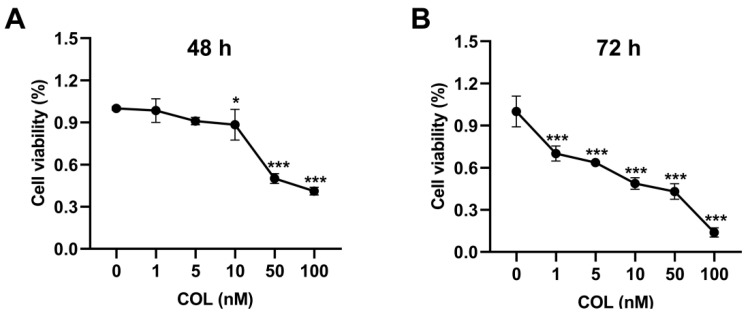
HK2 cell viability was assessed after (**A**) 48 h and (**B**) 72 h of exposure to colchicine at 0, 1, 5, 10, 50, and 100 nM. * *p* < 0.05, *** *p* < 0.001, *n* = 3.

A dose- and time-dependent decline in HK2 cell viability was observed upon colchicine exposure, with viability rates of 98.5%, 91.0%, and 88.4% at 1, 5, and 10 nM after 48 h, respectively, which further declined to 70.1%, 63.6%, and 48.7% at corresponding concentrations after 72 h (Figure 2A,B).

### 2.3. Differentially Expressed Genes (DEGs) in Colchicine-Treated HK2 Cells Determined via mRNA-Seq

**Figure 3:**
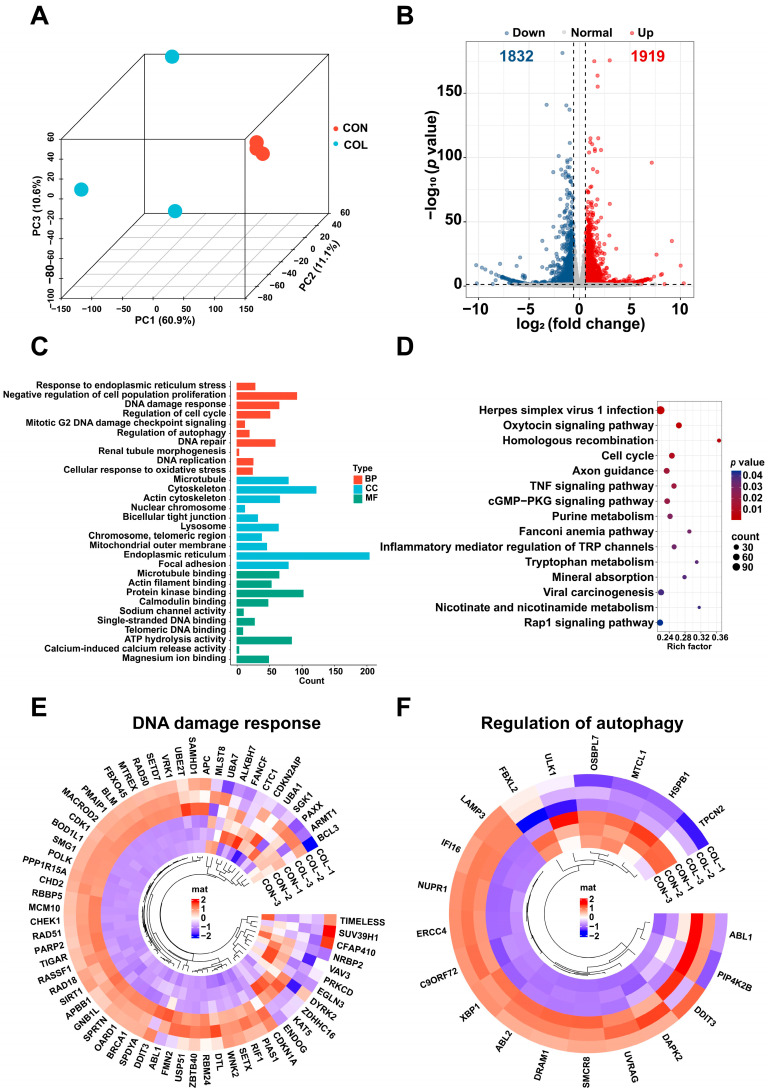
Transcriptomic profiling of HK2 cells treated with 5 nM colchicine for 72 h. (**A**) PCA plot showing the separation between the control and colchicine-treated groups. (**B**) Volcano plot of DEGs. (**C**) GO and (**D**) KEGG enrichment analyses of DEGs. Circular heatmaps of DEGs involved in (**E**) DNA damage response and (**F**) regulation of autophagy.

To elucidate the molecular mechanisms of colchicine-induced renal cell injury, an mRNA-seq analysis was performed on HK2 cells exposed to 5 nM colchicine for 72 h. A principal component analysis (PCA) demonstrated clear separation between the colchicine-treated and control groups (Figure 3A). A total of 3751 DEGs were identified based on the thresholds of *p* < 0.05 and |log_2_(fold change)| > 0.585, comprising 1919 upregulated and 1832 downregulated genes (Figure 3B). The DAVID database was used to perform GO and KEGG pathway enrichment analyses for the functional annotation of DEGs. The GO enrichment results mainly included DNA damage response, cellular response to oxidative stress, DNA repair, regulation of autophagy, and lysosome (Figure 3C). Meanwhile, the KEGG enrichment results mainly included cell cycle, homologous recombination, and purine metabolism (Figure 3D). A circular heatmap representation further revealed alterations in the expression of genes associated with DNA damage response and the regulation of autophagy (Figure 3E,F).

**Figure 4:**
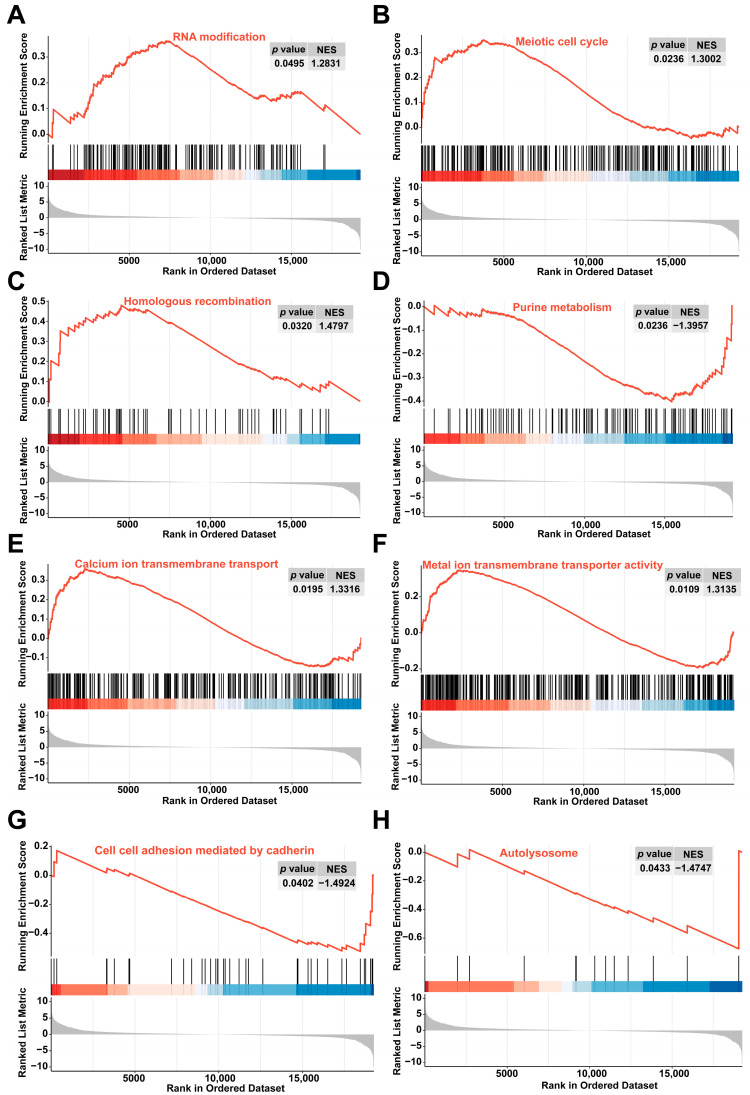
GSEA revealed enrichment in pathways related to (**A**) RNA modification, (**B**) meiotic cell cycle, (**C**) homologous recombination, (**D**) purine metabolism, (**E**) calcium ion transmembrane transport, (**F**) metal ion transmembrane transporter activity, (**G**) cell–cell adhesion mediated by cadherin, and (**H**) autolysosome.

Gene set enrichment analysis (GSEA) revealed that in the colchicine-treated groups compared with the controls, the RNA modification, meiotic cell cycle, homologous recombination, calcium ion transmembrane transport, and metal ion transmembrane transporter activity were significantly activated. Conversely, purine metabolism, cell–cell adhesion mediated by cadherin, and autolysosome pathways were suppressed (Figure 4).

### 2.4. Transcriptome-Wide Detection of m6A Modification After Colchicine Treatment of HK2 Cells

**Figure 5:**
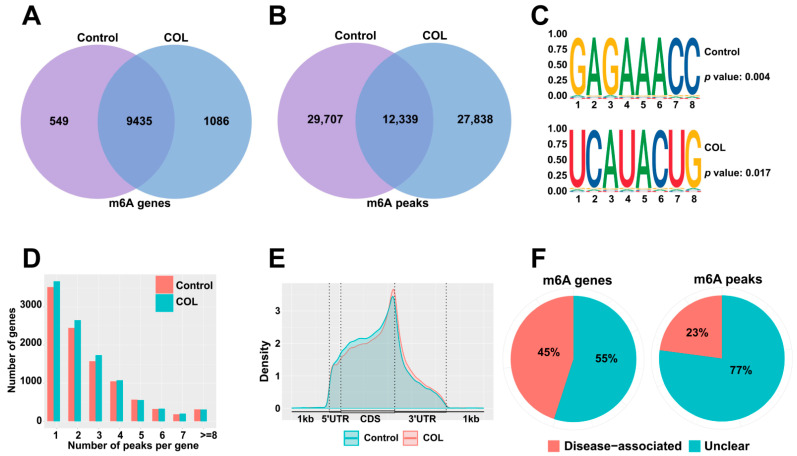
m6A modification patterns in HK2 cells after treatment with 5 nM colchicine for 72 h. Venn diagrams show the overlap of m6A (**A**) genes and (**B**) peaks between the control and colchicine-treated groups. (**C**) Sequence motifs of m6A peaks in the control and colchicine groups. (**D**) Distribution of the number of m6A peaks per gene in the control and colchicine-treated groups. (**E**) Density distribution of m6A-modified peaks in mRNA. (**F**) Pie charts depicting the proportion of disease-associated m6A genes and m6A peaks.

To investigate whether colchicine treatment induces alterations in m6A modifications in HK2 cells, a transcriptome-wide MeRIP-seq analysis was performed. In the control group, 9984 genes were identified with 42,046 m6A peaks detected, while the colchicine-treated group exhibited 40,177 m6A peaks among 10,521 m6A-modified genes (Figure 5A,B). The classical conserved m6A motif DRACH (D = A/G/U, R = A/G, H = A/C/U) was found to be significantly enriched in both the control and colchicine-treated groups according to the STREME analysis (Figure 5C). An analysis of peak distribution per transcript demonstrated that over 6000 transcripts harbored 1–3 m6A peaks in both groups, while only a minority contained more than 5 m6A modification peaks (Figure 5D). The m6A modifications were predominantly enriched in the coding sequence (CDS) regions of the transcripts (Figure 5E). Furthermore, an RMDisease analysis further revealed that 45% of m6A-modified genes and 23% of m6A peaks are involved in diseases (Figure 5F).

### 2.5. Integration of mRNA-Seq and MeRIP-Seq

**Figure 6:**
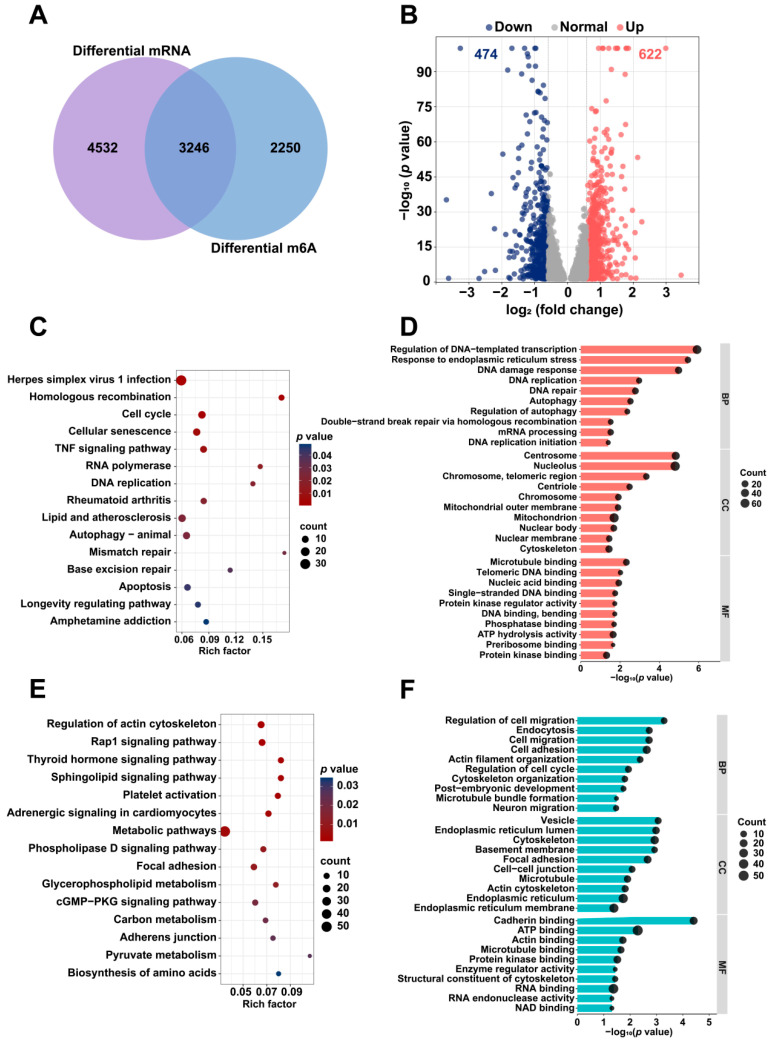
Differential mRNA expression and m6A modification analysis in HK2 cells treated with 5 nM colchicine for 72 h. (**A**) Venn diagram illustrating the overlap between DEGs and m6A-modified genes. (**B**) Volcano plot depicting the distribution of DEGs. Representative (**C**) KEGG and (**D**) GO enrichment pathways for upregulated genes following colchicine treatment. Representative (**E**) KEGG and (**F**) GO enrichment pathways for downregulated genes following colchicine treatment. The DEGs and differential m6A peaks were identified using *p* < 0.05.

Using a threshold of *p* < 0.05, the MeRIP-seq and mRNA-seq analyses identified 5496 differentially m6A-modified mRNAs and 7778 DEGs between the colchicine-treated and control groups. The intersection between differentially m6A-modified mRNAs and DEGs revealed 3246 overlapping genes (Figure 6A). With |log_2_(fold change)| > 0.585 as the selection criterion, 1096 DEGs were identified, comprising 622 upregulated and 474 downregulated genes (Figure 6B). A functional enrichment analysis was conducted using the DAVID database. The KEGG pathway analysis revealed that the upregulated genes were predominantly associated with the cell cycle, autophagy-animal, and DNA replication, while the downregulated genes were mainly enriched in pathways for the regulation of actin cytoskeleton, focal adhesion, and biosynthesis of amino acids. According to GO enrichment analysis, the upregulated genes showed significant enrichment in DNA damage response, DNA repair, autophagy, and mitochondrion, while the downregulated genes were enriched in cell adhesion, post-embryonic development, cell–cell junction, and ATP binding (Figure 6C–F).

**Figure 7:**
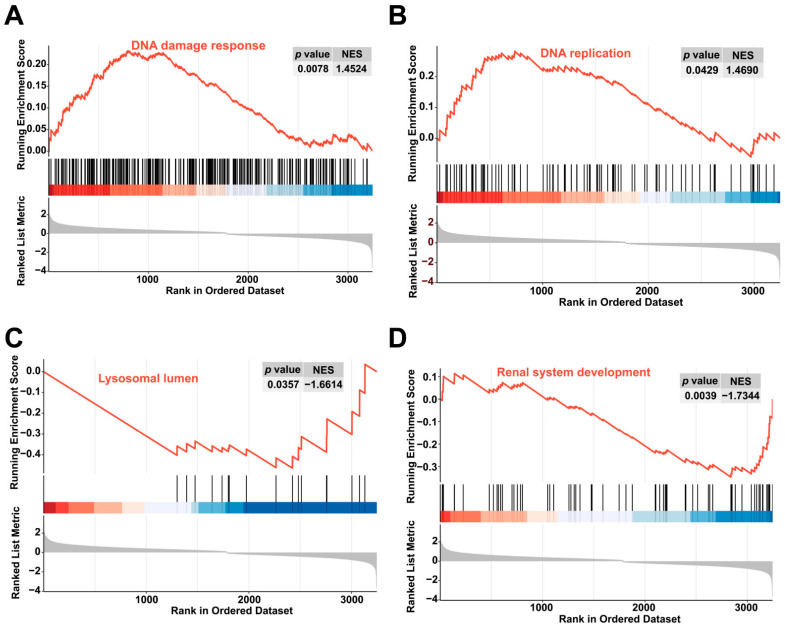
GSEA revealed enrichment in the (**A**) DNA damage response, (**B**) DNA replication pathways, (**C**) lysosomal lumen, and (**D**) renal system development pathways.

The GSEA was performed to identify the signaling pathways affected by colchicine exposure. Relative to the control group, the colchicine treatment groups activated the DNA damage response and DNA replication pathways, while inhibiting renal system development and lysosomal lumen pathways (Figure 7).

### 2.6. Molecular Interactions and MD Simulations Between Colchicine and Differentially Expressed m6A Modification Regulators

The mRNA-seq and MeRIP-seq data were utilized to assess both the levels of m6A methylation regulator expression and m6A modification to identify potential regulators, as shown in Table 1 and Table S1. *ZC3H13*, *YTHDC2*, *METTL14*, *METTL5*, *CBLL1*, *VIRMA*, *YTHDF1*, *FTO*, *WTAP*, *FMR1*, and *METTL3* exhibited significantly upregulated mRNA expression levels, whereas *IGF2BP3*, *HNRNPC*, *HNRNPA2B1*, *RBM15B*, and *IGF2BP2* showed significantly downregulated mRNA expression levels.

**Figure 8:**
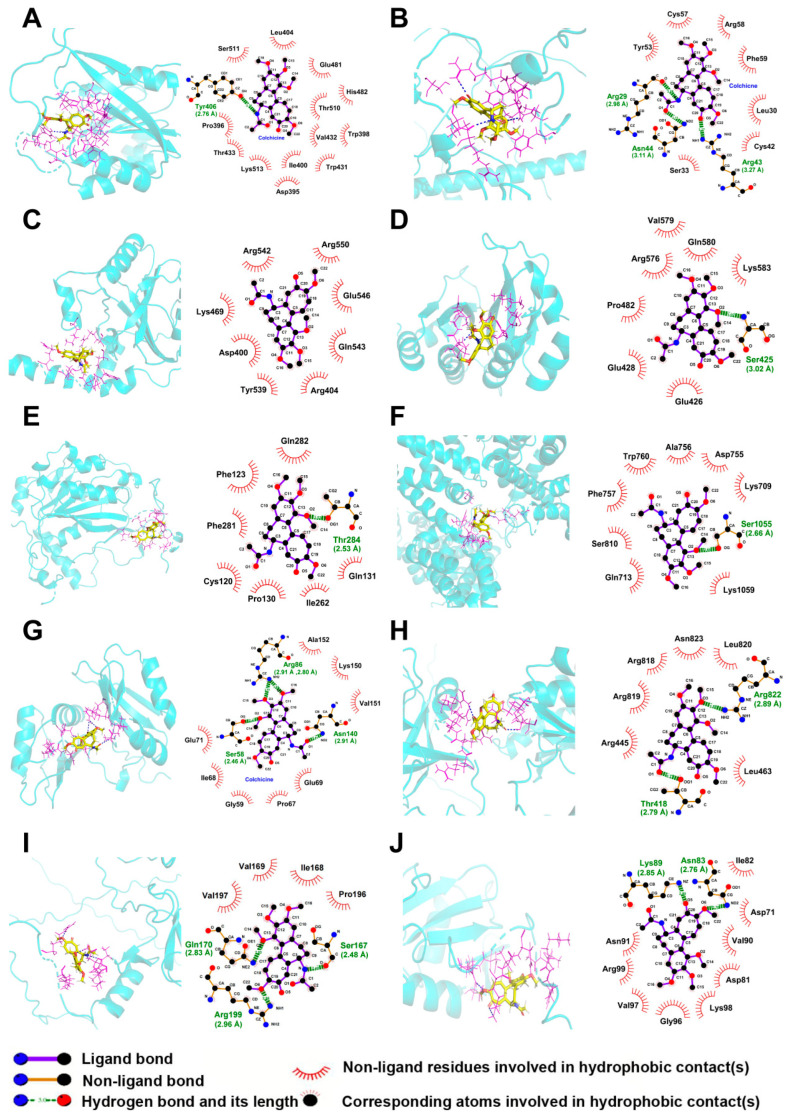
Three-dimensional and two-dimensional molecular docking models illustrated the interactions between colchicine and differentially expressed m6A regulators, including (**A**) METTL3, (**B**) ZC3H13, (**C**) YTHDF1, (**D**) IGF2BP2, (**E**) METTL14, (**F**) VIRMA, (**G**) IGF2BP3, (**H**) RBM15B, (**I**) CBLL1, and (**J**) HNRNPC.

**Figure 9:**
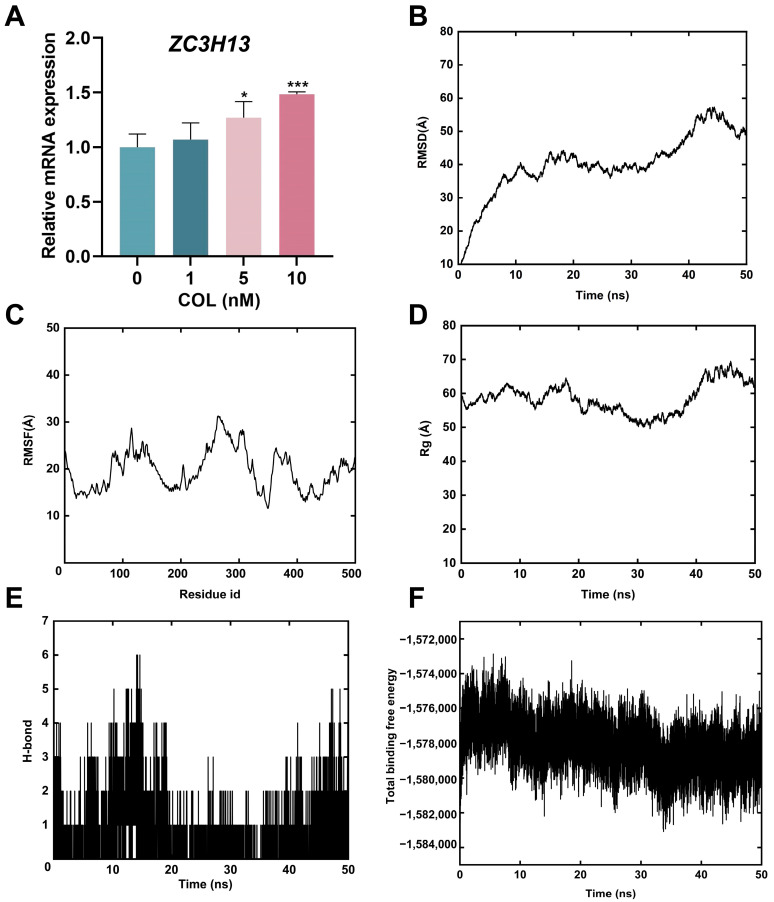
(**A**) Analysis of *ZC3H13* mRNA expression via RT-qPCR. (**B**) RMSD of the backbone atoms. (**C**) RMSF of amino acids for complexes. (**D**) Rg of the complex as a function of time during the simulation. (**E**) Hydrogen bonds formed by amino acids in the complexes. (**F**) Total binding free energy of the colchicine–ZC3H13 complex during MD simulations. * *p* < 0.05, *** *p* < 0.001, *n* = 3.

The molecular interactions between colchicine and differentially expressed m6A regulators were assessed to identify potential targets of colchicine. The total score derived from SYBYL-X 2.0 was based on van der Waals forces, hydrogen bonds, hydrophobic interactions, receptor–ligand geometric matching, and entropic changes. The predicted interactions between colchicine and the m6A regulators METTL3, ZC3H13, YTHDF1, IGF2BP2, METTL14, VIRMA, IGF2BP3, RBM15B, CBLL1, and HNRNPC, along with their corresponding total scores, are presented in Table 2 and Figure 8. A total score greater than 5 indicated stable binding between the colchicine and the m6A regulators. mRNA-seq analysis and molecular docking indicated that ZC3H13 was a potential molecular target of colchicine. Reverse transcription quantitative polymerase chain reaction (RT-qPCR) analysis demonstrated an upregulation of *ZC3H13* expression following colchicine treatment (Figure 9A). MD simulations were conducted to further explore the interaction between colchicine and ZC3H13.

The RMSD of the colchicine–ZC3H13 complex gradually reached a plateau during the MD simulation (Figure 9B), and the RMSF analysis quantified the residue fluctuations (Figure 9C). The Rg reflected the compactness of the protein structure during the simulation. As shown in Figure 9D, no notable changes in Rg were observed throughout the simulation, indicating that the protein structure maintained a high degree of compactness, suggesting its relative stability. Additionally, hydrogen bonds remained stable throughout the entire simulation process (Figure 9E). The total binding free energy began to stabilize 10 ns after the simulation started, indicating that the binding between ZC3H13 and colchicine gradually became stable (Figure 9F). The MD simulations indicated that ZC3H13 and colchicine could bind together stably.

### 2.7. Interactions Between Differentially Expressed m6A Modification Regulators and DNA Damage and Autophagy-Related Genes

**Figure 10:**
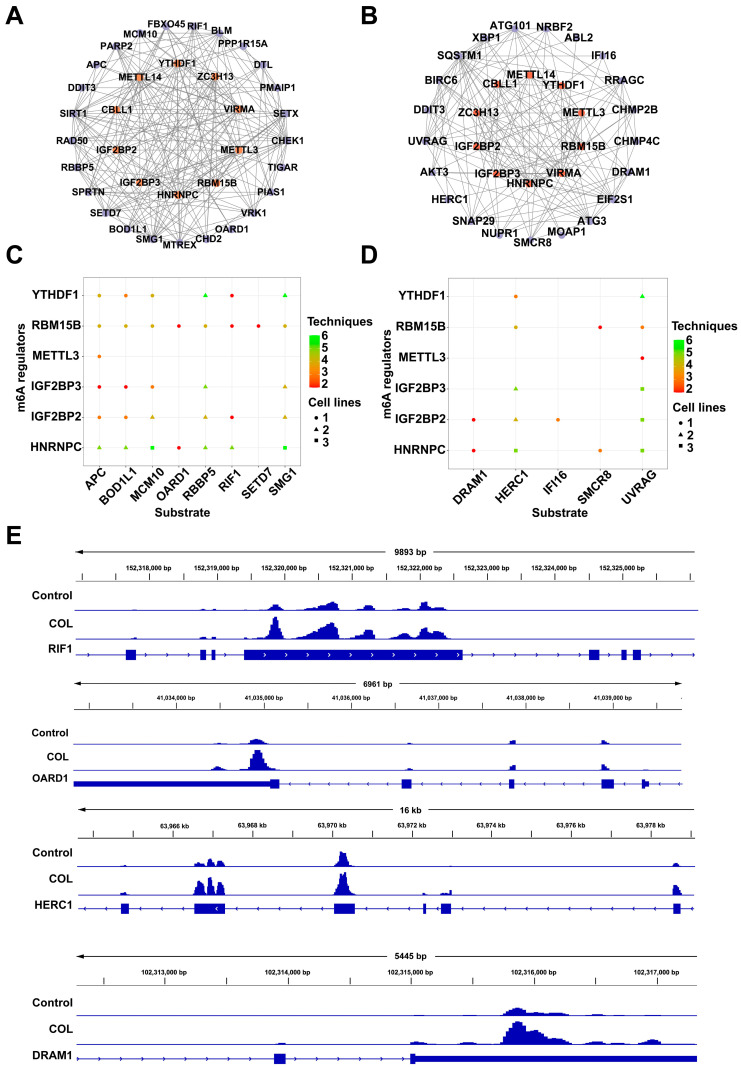
Interaction networks linking m6A regulators with (**A**) DNA damage response genes and (**B**) autophagy-related genes. Substrate prediction for the m6A regulators based on genes from (**C**) DNA damage response and (**D**) autophagy-related pathways. (**E**) Levels of m6A modification on *RIF1*, *OARD1*, *HERC1*, and *DRAM1* as observed via IGV.

A functional annotation based on the GO and KEGG databases indicated that pathways associated with DNA damage response and autophagy were significantly enriched. The STRING database was used to explore the potential regulatory interactions between differentially expressed m6A regulators and target genes in the related signaling pathways, and protein–protein interaction (PPI) networks were subsequently constructed. Among the DNA damage response related genes, *CHEK1*, *BLM*, *PARP2*, *SIRT1*, and *DTL* exhibited degrees of 8, 6, 5, 5, and 5, respectively. For the autophagy-related genes, *SQSTM1*, *CHMP2B*, *ATG101*, *UVRAG*, and *SMCR8* showed degrees of 12, 7, 7, 6, and 5, respectively (Figure 10A,B). Substrates potentially regulated by m6A methylation factors were identified through CLIP-seq analysis. The m6A regulators YTHDF1, RBM15B, METTL3, IGF2BP3, IGF2BP2, and HNRNPC were selected to assess their regulatory associations with DNA damage response and autophagy-related genes (Figure 10C,D). Visualization using integrative genomics viewer (IGV) revealed increased m6A modification on DNA damage response genes *RIF1* and *OARD1*, as well as autophagy-related genes *DRAM1* and *HERC1*, in the colchicine-treated group (Figure 10E).

### 2.8. DNA Damage Induced by Colchicine

**Figure 11:**
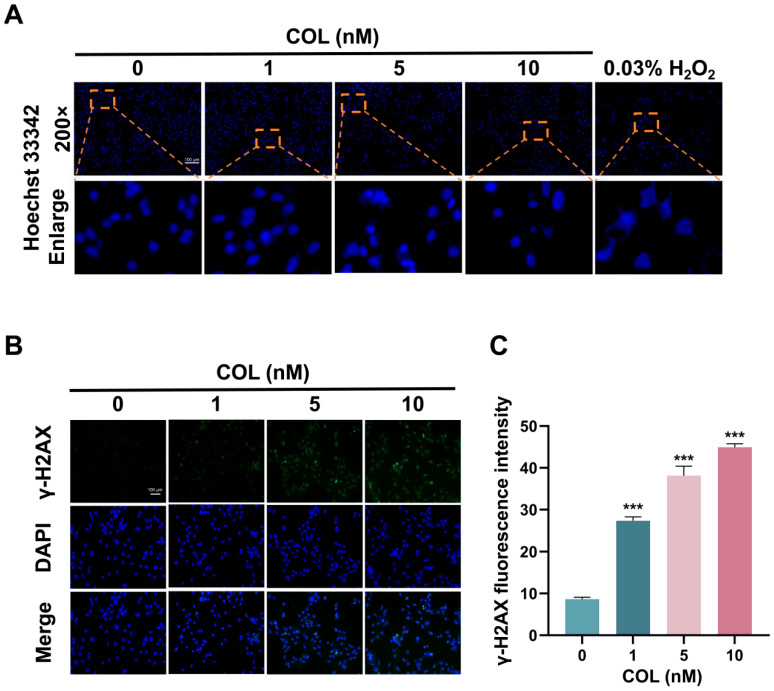
The impact of colchicine on DNA damage in HK2 cells following 72 h of exposure. (**A**) Nuclear fragments visualized with Hoechst 33342 staining. Orange dashed boxes indicated regions shown at higher magnification. (**B**) Representative images of γ-H2AX immunofluorescence intensity. (**C**) Quantitative analysis of γ-H2AX immunofluorescence. *** *p* < 0.001, *n* = 3. Scale bar = 100 μm.

Hoechst 33342, a cell-permeable dye, specifically bound to the minor groove of double-stranded DNA and exhibited blue fluorescence. As shown in Figure 11A, 5 and 10 nM colchicine treatment induced chromatin condensation and nuclear fragmentation, indicating significant DNA damage compared with that in the control.

The cumulative effect of colchicine on the accumulation of phosphorylated H2AX (γ-H2AX) in HK2 cells was measured to evaluate DNA damage. After treating HK2 cells with 0, 1, 5, and 10 nM of colchicine for 72 h, the content of γ-H2AX in the cells was measured. Following DNA injury, histone H2AX was phosphorylated to generate γ-H2AX, which then accumulated at the damaged sites and formed a fluorescence-detectable focus. Upon exposure to 1, 5, and 10 nM of colchicine, the average fluorescence intensities were elevated 3.2-, 4.4-, and 5.2-fold relative to the baseline intensity observed with 0 nM (Figure 11B,C).

### 2.9. DNA Damage and Autophagy in HK2 Cells Induced by Colchicine

**Figure 12:**
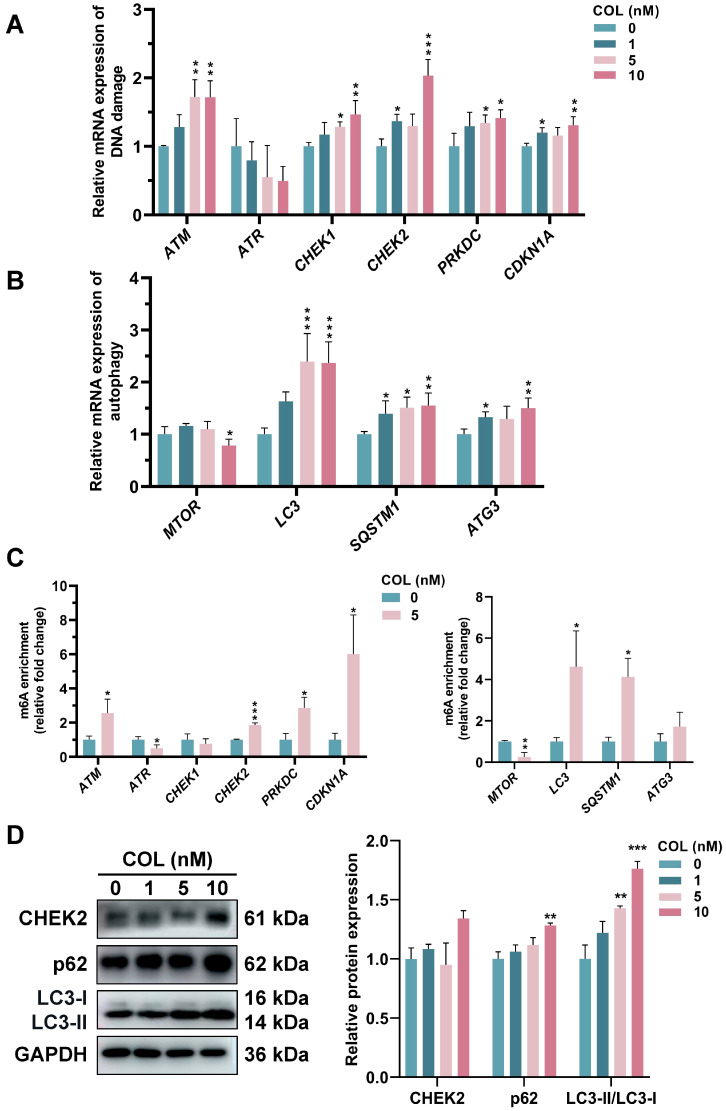
RT-qPCR analysis of mRNA expression related to (**A**) DNA damage and (**B**) autophagy. (**C**) MeRIP-qPCR analysis of m6A levels in DNA damage- and autophagy-related genes following 72 h treatment with 5 nM colchicine. (**D**) The expression of CHEK2, LC3, and p62 proteins was assessed in colchicine-treated HK2 cells. * *p* < 0.05, ** *p* < 0.01, *** *p* < 0.001, *n* = 3.

The mRNA expression levels associated with DNA damage and autophagy in the HK2 cells after colchicine treatment are presented in Figure 12A,B. In the genes associated with DNA damage, the expression levels of *ATM*, *CHEK1*, *CHEK2*, *PRKDC*, and *CDKN1A* were significantly elevated. Treatment with 10 nM colchicine resulted in a 1.7-fold increase in *ATM* expression and a 2.0-fold increase in *CHEK2* expression compared with those in the control group. Among the autophagy-related genes, *MTOR* was downregulated, while *LC3*, *SQSTM1* and ATG3 were upregulated. Treatment with 10 nM colchicine resulted in a 2.4-fold upregulation of the autophagy marker *LC3* compared with that in the control. Moreover, MeRIP-qPCR showed altered m6A modification levels in these genes (Figure 12C). A Western blot analysis demonstrated colchicine-induced DNA damage due to the increase in CHEK2 in the 10 nM treatment group compared to controls. LC3-II/LC3-I, an indicator of autophagosome biogenesis, exhibited a dose-dependent increase, with a fold change of 1.76 in 10 nM treatment group, with a 28.2% upregulation in p62 levels compared with that in the control group (Figure 12D). An increase in the LC3-II/LC3-I ratio, along with an increase in p62 expression, indicated colchicine might impair autophagy flux by blocking autophagosome–lysosome fusion or lysosomal degradation, leading to the accumulation of LC3-II and p62.

### 2.10. Knockdown of ZC3H13 Gene Expression Reduced the Induction by Colchicine in HK2 Cells

**Figure 13:**
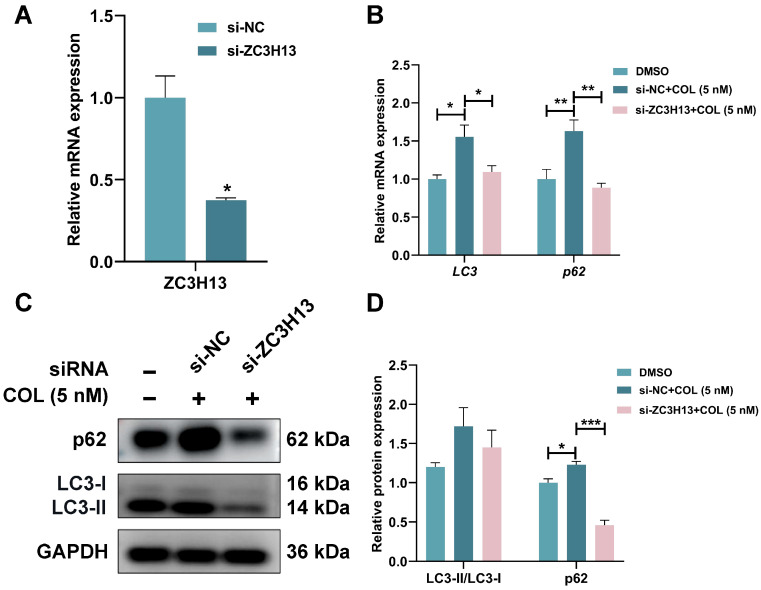
(**A**) The knockdown efficiency of ZC3H13 was assessed following transfection with siRNA-ZC3H13 and treatment with colchicine, followed by (**B**) RT-qPCR analysis of LC3 and p62 mRNA expression, (**C**,**D**) Western blot and quantitative analysis of p62 and LC3 proteins. * *p* < 0.05, ** *p* < 0.01, *** *p* < 0.001, *n* = 3.

To investigate the potential role of ZC3H13 in colchicine-induced nephrotoxicity, ZC3H13 expression was downregulated in HK2 cells through siRNA transfection, thereby enabling examination of its functional impact on colchicine responses. RT-qPCR analysis demonstrated that ZC3H13 knockdown significantly attenuated colchicine-mediated phenotypic alterations in HK2 cells (Figure 13A,B). Colchicine-treated HK2 cells transfected with si-ZC3H13 decreased LC3-II/LC3-I ratio, along with p62 protein levels (*p* < 0.001, Figure 13C,D). These findings suggested a critical involvement of ZC3H13 in colchicine-induced autophagic activation within HK2 cells.

## 3. Discussion

Colchicine, a bioactive alkaloid derived from plants of the genus *Colchicum*, exhibits remarkable pharmacological and toxicological properties. Its pharmacological effects have been extensively documented, including anti-inflammatory activity, cardioprotective effects, anti-proliferative actions, and metabolic regulation. The standard clinical dosage of colchicine for adults was established at 0.5 mg per day. O. Chappey et al. documented in their study “Colchicine concentration in leukocytes of patients with familial Mediterranean fever” that adult patients receiving 0.5 mg daily exhibited plasma concentrations of 0.33 nM [16]. Our experimental concentrations of 1, 5, and 10 nM represented 3-, 15-, and 30-fold increases, respectively, over these clinical exposure levels, thus providing a preclinical toxicological reference. However, its toxicological mechanisms diverged markedly from its pharmacological mechanisms. The molecular pathogenesis of colchicine toxicity involved a multifaceted cascade, including reduced β-tubulin acetylation and end-binding protein 1 displacement, disrupting microtubule dynamics and compromising cellular polarity and transport [17]. Mitochondrial dysfunction emerged via suppressed cytochrome C oxidase subunit IV expression, concomitant with a fourfold increase in reactive oxygen species and mtDNA leakage [18]. Notably, 10 nM colchicine activated PINK1/Parkin-mediated mitophagy, yet sustained exposure disrupted mitochondrial quality control [19]. At 100 nM, the HK2 cells underwent ferroptosis characterized by GPX4 downregulation and ACSL4 upregulation, which was reversible via iron chelation [20]. While colchicine toxicity was well-documented, its precise mechanisms remained elusive. Emerging evidence has suggested that DNA damage, autophagy, and m6A RNA modification were implicated in colchicine-induced toxicity. In this study, HK2 cells were employed to elucidate the toxicological mechanisms of colchicine from the perspectives of DNA damage and autophagy.

DNA damage was a common cellular stress response triggered by various endogenous metabolic byproducts or exogenous environmental insults and was involved in regulating a wide range of physiological and pathological processes. Double-strand breaks (DSBs), identified as the most harmful DNA damage type, compromised genomic integrity and stability. γ-H2AX upregulation was one of the earliest cellular events to occur in response to DSBs and was used as a biomarker to evaluate the presence and extent of DNA damage [21]. In the present study, treatment of the HK2 cells with colchicine led to increased nuclear fragmentation and a significant upregulation of γ-H2AX expression, indicating that colchicine induced DNA damage. In addition to serving as a marker of DNA damage, γ-H2AX was also involved in chromatin remodeling and the regulation of DNA repair processes [22]. Phosphatidylinositol 3-kinase-related kinase (PIKK) family members, including ATM and DNA-PKcs, mediated DNA damage response activation [23]. Subsequently, CHEK1 and CHEK2 orchestrated cell cycle arrest and coordinated DNA repair. Tumor protein p53 and p21 suppressed the cell cycle to facilitate DNA repair [24]. RT-qPCR analysis revealed that colchicine treatment significantly upregulated key DDR genes in HK2 cells, including *ATM*, *DNA-PKcs*, *CHEK1*, *CHEK2*, and *CDKN1A*, indicating colchicine-induced DNA damage. When DNA damage was not efficiently repaired, cells activated autophagy pathways to cope with persistent stress. Liu et al. demonstrated that under ultraviolet irradiation or methyl methanesulfonate treatment, the DNA damage response mediated by the ATR-Chk1 pathway could lead to pronounced activation of autophagy, contributing to cell death [25]. These findings suggested that the accumulation of DNA damage not only caused cell cycle arrest but could also provoke abnormal or excessive autophagic responses, thereby promoting progression toward cell death.

Autophagy is a process by which cells degrade and recycle proteins and organelles to maintain intracellular homeostasis [26,27]. The autophagy process is regulated by mTOR inhibition and AMPK activation, relying on a series of autophagy-related proteins [26]. Autophagosomes form and engulf damaged organelles, excessively accumulated proteins, and pathogens, which are recognized and guided by the autophagy receptor p62/SQSTM1. P62 binds to the autophagosomal membrane, facilitating phagophore expansion and elongation, and promotes LC3-I transformation into LC3-II [28]. LC3-II facilitates phagophore extension and maturation [29]. Ultimately, fusion between autophagosomes and lysosomes leads to degradation [8]. Ashok Kumar et al. demonstrated that colchicine treatment induced autophagic vacuoles and LC3-I to LC3-II conversion, suggesting autophagy activation in HCT116 cells [30]. Notably, use of the autophagy inhibitor 3-MA notably decreased colchicine-induced cell death, demonstrating that autophagy played a role in colchicine-induced cell death. Studies demonstrated that colchicine-induced DNA damage triggered protective autophagy, which selectively degraded key regulatory proteins including Rnr1, USP14, CHK1, and NOXA, thereby directly or indirectly disrupting DNA repair mechanisms and modulating cell fate decisions such as proliferation, apoptosis, or senescence. In this study, RT-qPCR analysis revealed abnormal alterations in the expression of autophagy-related genes *MTOR*, *LC3*, *P62*, and *ATG3* in colchicine-treated HK2 cells. The protein expression levels of p62 and LC3-II were upregulated, as shown by Western blot analysis. Collectively, these results indicated that colchicine treatment induced autophagy.

m6A modification, the most prevalent epitranscriptomic modification in eukaryotic mRNAs, dynamically regulated RNA metabolism and participated in critical biological processes including DNA damage repair and autophagy activation [31]. In this study, GO and KEGG pathway enrichment analyses of DEGs and differentially methylated genes revealed predominant associations with DNA damage response and autophagy, consistent with colchicine-induced DNA damage and autophagic activation, suggesting m6A-mediated regulation of colchicine nephrotoxic effects. KEGG analysis further demonstrated that DEGs were enriched in cell cycle and homologous recombination pathways, while hypomethylated genes predominantly clustered in the post-embryonic developmental pathways, implying that the m6A-dependent modulation of colchicine results in antiproliferative effects. This study also identified inflammation-related pathways in DEGs and methylated genes, including TNF signaling and the inflammatory mediator regulation of TRP channels, aligning with the anti-inflammatory mechanisms of colchicine reported by Deftereos et al. [32]. Beyond colchicine, other herbal compounds have exhibited similar epigenetic regulatory mechanisms. For instance, Li et al. revealed a novel antihypertensive mechanism of rhynchophylline via SIRT3-dependent mitochondrial homeostasis modulation, validating SIRT3 agonists as potential therapeutic strategies. These findings collectively underscored the pivotal role of epitranscriptomic modifications in phytomedicine research [33].

## 4. Conclusions

This study demonstrated that colchicine modulates the expression of DNA damage- and autophagy-related genes while altering m6A modification levels in HK2 cells, indicating its role in renal cytotoxicity. The study indicated that γ-H2AX and DNA damage-related genes such as *ATM* and *CHEK1* were upregulated and that autophagy markers such as LC3-II and p62 were changed. Furthermore, m6A modifications were implicated in the regulation of DNA damage and autophagic pathways. These results revealed a mechanistic link among DNA damage, autophagy, and m6A modification in colchicine-induced nephrotoxicity.

## 5. Materials and Methods

### 5.1. Chemical Reagent, Cell Culture, and siRNA Transfection

Colchicine was purchased from Must Bio-Technology (Chengdu, China). Its chemical structure was characterized via ^1^H and ^13^C NMR spectroscopy at 400 MHz using a Bruker AVANCE III 400 MHz spectrometer (Bruker BioSpin, Rheinstetten, Germany). The purity was verified via HPLC analysis using a Dionex UltiMate 3000 system (Thermo Fisher Scientific, Waltham, MA, USA) equipped with a C18 column (4.6 mm × 250 mm, 5 μm). The human renal proximal tubular epithelial cells (HK2) were obtained from the American Type Culture Collection (ATCC) and cultured in DMEM containing 10% fetal bovine serum (Gibco, New York, NY, USA) and 1% penicillin–streptomycin. The cells were incubated at 37 °C with 5% CO_2_. Colchicine was dissolved in dimethyl sulfoxide (DMSO), and the final working concentration of DMSO in the cell culture experiments was 0.1%.

HK2 cells were seeded in 12-well plates and transfected with si-ZC3H13 (GenePharma, Shanghai, China) at approximately 60% confluency. For transfection, 17 μL buffer, 40 pmol si-ZC3H13, and 3 μL transfection reagent (GenePharma) were mixed gently to form complexes, which were then added to cells. RNA was extracted 48 h post-transfection to assess silencing efficiency. Cells were treated with 5 nM colchicine 36 h post-transfection for 72 h. The siRNA sequences used in this study are listed in Table S2.

### 5.2. Cell Viability Assay

The HK2 cells were plated in 96-well plates at an initial density of 3 × 10^3^ cells and cultured overnight in a 37 °C incubator (Thermo Fisher, Langenselbold, Germany) to allow for adherence. The cells were then treated with colchicine at concentrations of 0, 1, 5, 10, 50, and 100 nM for 48 h and 72 h. Subsequently, 10 μL of 5 mg/mL MTT solution was added to each well and incubated at 37 °C in the dark for 4 h. The supernatant was discarded, followed by the addition of 100 μL of DMSO to dissolve formazan crystals. A multifunctional microplate reader (BioTek, Santa Clara, CA, USA) was employed to measure absorbance at 490 nm.

### 5.3. PharmMapper Predicted Potential Targets of Colchicine

The molecular structure of colchicine was retrieved from the PubChem database and subjected to energy minimization to generate a mol_2_ file, which was subsequently submitted to the PharmMapper server for analysis. The resulting UniProt IDs were standardized using the UniProt database and converted to their corresponding gene symbols for subsequent enrichment analysis.

### 5.4. RNA Extraction from HK2 Cells

The HK2 cells were cultured in 6-well plates and treated with colchicine at 0, 1, 5, and 10 nM for 72 h. Then, TRIzol reagent (Invitrogen, Carlsbad, CA, USA) was added to lyse the cells. A Qubit 3.0 fluorometer (Thermo Fisher, Waltham, MA, USA) was employed to determine the RNA concentration.

### 5.5. High-Throughput Sequencing

Total RNA was extracted from the HK2 cells following 72 h of exposure to 5 nM colchicine. mRNA-seq and MeRIP-seq were performed by Seqhealth (Wuhan, China). The mRNA was sheared into fragments of approximately 100–200 nt, with one portion used as the input group, and the remaining fragments were subjected to immunoprecipitation (IP) using an m6A-specific antibody to enrich for the IP group [34,35]. Libraries for both groups were constructed using the unique molecular identifier strategy and sequenced on the Novaseq 6000 sequencer (Illumina, San Diego, CA, USA).

### 5.6. Bioinformatics Analysis

Trim Galore was used to perform quality control and adapter trimming of the raw sequencing data. Alignment of the cleaned reads to the hg19 human genome reference was performed using Hisat2 [36]. StringTie was utilized to quantify transcript expression, and DESeq was employed to detect DEGs. The package ExomePeak2 was employed to identify m6A methylation peaks and differential methylation [37]. STREME was utilized to characterize m6A-associated motif sequences [38]. The disease relevance of m6A methylation sites was assessed using RMDisease [39]. The visualization of m6A methylation profiles was performed with the IGV software (version 2.14). The mRNA-seq and MeRIP-seq datasets were submitted to the GEO database (ID: GSE295178).

### 5.7. Molecular Docking and MD Simulation

To investigate the binding interactions between colchicine and differentially expressed m6A regulators, molecular docking was carried out using SYBYL-X 2.0. The protein structures were retrieved from the Uniprot and PDB databases and then preprocessed using PYMOL. This preprocessing involved the removal of excess ligands, the deletion of redundant peptide chains, and elimination of water molecules.

Using the Python package XPONGE (version 1.3.4), the complex was constructed [40]. Simulations were performed using the FF14SB united-atom force field. The systems were solvated with the SPC/E water model and neutralized with K^+^ and Cl^−^, and periodic boundary conditions were applied. Energy minimization was performed with a tolerance of 5.0 kJ/mol, followed by 100 ps NVT equilibration at 300 K and 100 ps NPT simulation at 1 bar. Each system underwent a 50 ns MD simulation. To assess binding stability in a dynamic context, a range of MD trajectory analyses were applied to the simulation results, including the Rg, the number of hydrogen bonds, and the total binding free energy.

### 5.8. DNA Damage Detected via Nuclear Staining

Following 72 h of colchicine treatment in a 24-well plate, the HK2 cells were treated with 0.03% H_2_O_2_ for 20 min as a DNA damage positive control. Hoechst 33342 (Beyotime, Shanghai, China) staining of the HK2 cells was carried out at 37 °C for 10 min under dark conditions. Nuclear morphology was examined using a fluorescence microscope (Zeiss, Oberkochen, Germany) with an excitation wavelength of 365 nm.

### 5.9. Fluorescence Analysis of γ-H2AX

The HK2 cells were exposed to 0, 1, 5, and 10 nM colchicine for 72 h, followed by fixation using 4% paraformaldehyde. The cells were incubated with γ-H2AX rabbit monoclonal antibody for 1 h at 25 °C. The nuclei were subsequently stained with DAPI following 1 h of incubation with a fluorescently labeled secondary antibody. The cells were observed and imaged under an inverted fluorescence microscope (Zeiss), and the fluorescence intensity was measured using ImageJ software (version 1.53t). The experiment were replicated three times independently.

### 5.10. Reverse Transcription Quantitative PCR (RT-qPCR) and MeRIP-qPCR Analysis

RNA was reverse-transcribed into cDNA using a reverse transcription premix kit (Accurate Biology, Changsha, China). RT-qPCR was performed using SYBR Green Pro Taq HS Premix II on the AriaMX Real-Time PCR System (Agilent, Santa Clara, CA, USA). Relative mRNA expression levels were determined using the 2^−ΔΔCt^ method, with *GAPDH* as the internal reference gene. The primer sequences are listed in Table 3.

The HK2 cells were treated with 5 nM colchicine for 72 h. Total RNA was extracted, and a portion was reserved as the input group. Protein A/G magnetic beads (Selleck, Houston, TX, USA) were pre-incubated with anti-m6A antibody (Proteintech, Wuhan, China) or IgG control in an immunoprecipitation buffer at room temperature for 1 h. The remaining RNA was incubated with the antibody–bead complexes in immunoprecipitation buffer containing RNasin at 4 °C overnight. After incubation, the complexes were eluted with an elution buffer at 55 °C for 1 h to release any bound RNA. The immunoprecipitated RNA was then extracted using the TRIzol method. Both input and immunoprecipitated RNA samples were reverse-transcribed into cDNA, followed by an RT-qPCR analysis.

### 5.11. Western Blot Analysis

The HK2 cells were treated with 0, 1, 5, and 10 nM colchicine for 72 h. Total protein was extracted with a RIPA buffer containing inhibitors, centrifuged at 12,000× *g* for 15 min. The proteins were transferred to PVDF membranes after separation via 15% SDS-PAGE. Following blocking with 5% skim milk, the membranes were incubated at 4 °C overnight with primary antibodies, including LC3 (Selleck, Houston, TX, USA), CHEK2 and p62 (Proteintech, Wuhan, China). Secondary antibodies (Proteintech) were applied for 90 min. Signals were detected using an Amersham Imager 680 System (Cytiva, Marlborough, MA, USA) and analyzed using ImageJ. The experiments were replicated three times independently.

### 5.12. Statistical Analysis

One-way analysis of variance (ANOVA) was conducted on the experimental data using the SPSS 26 software (IBM, New York, NY, USA). Results were presented as mean ± standard deviation, with statistical significance determined at *p* < 0.05. Each experiment was repeated at least three times independently.

## Figures and Tables

**Table 1:** The mRNA expression levels of m6A regulators in colchicine-treated HK2 cells.

Gene	Regulation	Base Mean	Log_2_Fold Change	*p* Value	*p* adj
*IGF2BP3*	reader	3355	−0.61	1.20 × 10^−23^	4.24 × 10^−22^
*ZC3H13*	writer	14,808	0.58	1.99 × 10^−15^	3.44 × 10^−14^
*YTHDC2*	reader	1442	0.49	6.04 × 10^−12^	7.17 × 10^−11^
*METTL14*	writer	2091	0.53	1.68 × 10^−10^	1.70 × 10^−9^
*HNRNPC*	reader	27,180	−0.45	5.73 × 10^−9^	4.80 × 10^−8^
*METTL5*	writer	1070	0.61	6.25 × 10^−9^	5.22 × 10^−8^
*CBLL1*	writer	1534	0.43	7.25 × 10^−9^	6.01 × 10^−8^
*HNRNPA2B1*	reader	80,606	−0.30	1.66 × 10^−8^	1.32 × 10^−7^
*RBM15B*	writer	3167	−0.29	4.09 × 10^−5^	1.95 × 10^−4^
*VIRMA*	writer	4732	0.25	2.01 × 10^−4^	8.37 × 10^−4^
*YTHDF1*	reader	2182	0.26	2.09 × 10^−3^	6.93 × 10^−3^
*FTO*	eraser	1586	0.37	3.19 × 10^−3^	1.01 × 10^−2^
*WTAP*	writer	5863	0.25	5.72 × 10^−3^	1.69 × 10^−2^
*FMR1*	reader	4443	0.17	7.15 × 10^−3^	2.06 × 10^−2^
*METTL3*	writer	1593	0.21	1.95 × 10^−2^	4.93 × 10^−2^
*IGF2BP2*	reader	9542	−0.23	2.75 × 10^−2^	6.61 × 10^−2^
*ALKBH5*	eraser	3141	0.08	2.46 × 10^−1^	3.86 × 10^−1^
*IGF2BP1*	reader	3971	0.12	3.50 × 10^−1^	5.04 × 10^−1^
*RBM15*	writer	1166	0.30	5.49 × 10^−1^	6.96 × 10^−1^
*YTHDF3*	reader	1976	0.19	6.70 × 10^−1^	7.92 × 10^−1^
*YTHDC1*	reader	12,503	0.08	6.90 × 10^−1^	8.06 × 10^−1^
*YTHDF2*	reader	2483	0.02	9.28 × 10^−1^	9.61 × 10^−1^

**Table 2:** The interactions between colchicine and m6A regulators.

Protein	PDB/ Uniprot ID	Total Score	Crash	Polar	H-Bond Number	Residues Involved in H-Bond Formation	Hydrophobic Contact Number	Residues Involved in Hydrophobic Contacts
METTL3	5IL2	7.783	−3.022	1.063	1	Tyr406	13	Ser511, Leu404, Glu481, His482, Thr510, Val432, Trp398, Trp431, Ile400, Asp395, Lys513, Thr433, Pro396
ZC3H13	Q5T200	7.169	−1.561	2.834	3	Arg29, Asn44, Arg43	7	Tyr53, Cys57, Arg58, Phe59, Leu30, Cys42, Ser33
YTHDF1	8BS4	6.808	−2.435	0.001	0	-	8	Arg542, Arg550, Glu546, Gln543, Arg404, Tyr539, Asp400, Lys469
IGF2BP2	6ROL	6.304	−1.593	2.453	1	Ser425	7	Arg576, Val579, Gln580, Lys583, Gu426, Glu428, Pro482
METTL14	5IL2	5.999	−1.938	1.882	1	Thr284	7	Gln131, Ile262, Pro130, Cys120, Phe281, Phe123, Gln282
VIRMA	7YG4	5.910	−0.973	3.119	1	Ser1055	8	Lys709, Asp755, Ala756, Trp760, Phe757, Ser810, Gln713, Lys1059
IGF2BP3	6FQ1	5.689	−2.254	2.940	4	Asn140, Ser58, Arg86 (2 H-bonds)	7	Glu71, Ile68, Gly59, Pro67, Glu69, Val151, Lys150, Ala152
RBM15B	Q8NDT2	5.478	−1.434	3.971	2	Arg822, Thr418	6	Arg445, Arg819, Arg818, Asn823, Leu820, Leu463
CBLL1	Q75N03	5.077	−3.395	2.878	3	Gln170, Ser167, Arg199	4	Val197, Val169, Ile168, Pro196
HNRNPC	2MXY	5.016	−1.809	2.157	2	Lys89, Asn83	9	Ile82, Arg99, Asp71, Asn91, Val90, Asp81, Lys98, Gly96, Val97

**Table 3:** Sequences of primers used in RT-qPCR.

Gene	Forward Primer (5′-3′)	Reverse Primer (5′-3′)
*ATM*	GCTGTGGTGGAGGGAAGATGTTAC	CCTGCCTGGCGTGTTGATGAG
*ATR*	CACCACCAGACAGCCTACAATGC	CCAGAGCCACTTTGCCCTTTCC
*CHEK1*	CTGCCACATGATCGGACCATCG	GAGAATCGCTTGAACCCAGGAGAC
*CHEK2*	CCAGCCAGTCCTCTCACTCCAG	GGTTCTTGGTCCTCAGGTTCTTGG
*PRKDC*	AGTGAGCCAGCCTGCCTTG	CACCTTCTCTGAATCCTCTGAACTG
*CDKN1A*	TCCAGCGACCTTCCTCATCCAC	TCCATAGCCTCTACTGCCACCATC
*MTOR*	GAGATACGCTGTCATCCCTTTA	CTGTATTATTGACGGCATGCTC
*LC3*	GCCTTCTTCCTGCTGGTGAACC	TCCTCGTCTTTCTCCTGCTCGTAG
*SQSTM1*	TGATTGAGTCCCTCTCCCAGATGC	CCGCTCCGATGTCATAGTTCTTGG
*ATG3*	CGGTGCAAACAGATGGAATATT	GTGTGATCTCTTTAACGGCTTC
*ZC3H13*	GAGGTGACAGAAGCAGAGCATAC	GGCGGTGGAGGAGGAAGAAG
*GAPDH*	TGACATCAAGAAGGTGGTGAAGCAG	GTGTCGCTGTTGAAGTCAGAGGAG

*ATM:* ataxia telangiectasia mutated; *ATR*: ataxia telangiectasia and rad3-related protein; *CHEK1*: checkpoint kinase 1; *CHEK2*: checkpoint kinase 2; *PRKDC*: protein kinase, DNA-activated, catalytic subunit; *CDKN1A*: cyclin-dependent kinase inhibitor 1A; *MTOR*: mechanistic target of rapamycin; *LC3*: microtubule-associated protein 1A/1B-light chain 3; *SQSTM1*: sequestosome 1; *ATG3*: autophagy related 3; *ZC3H13*: Zinc finger CCCH-type containing 13; *GAPDH*: glyceraldehyde-3-phosphate dehydrogenase.

## Data Availability

The original contributions presented in this study are included in this article and supplementary material. Further inquiries can be directed to the corresponding author.

## Supplementary Materials

The following supporting information can be downloaded at https://www.mdpi.com/article/10.3390/toxins17080408/s1, Table S1: The m6A modification levels of regulators in colchicine-treated HK2 cells; Table S2: Sequence of siRNA-ZC3H13.

## Abbreviations

The following abbreviations are used in this manuscript:

mRNA-seq	mRNA sequencing
MeRIP-seq	Methylated RNA immunoprecipitation sequencing
MD	Molecular dynamics
NMR	Nuclear magnetic resonance
HPLC	High-performance liquid chromatography
GO	Gene Ontology
KEGG	Kyoto Encyclopedia of Genes and Genomes
DEGs	Differentially expressed genes
PCA	Principal component analysis
GSEA	Gene set enrichment analysis
CDS	Coding sequence
RT-qPCR	Reverse transcription quantitative polymerase chain reaction
PPI	Protein–protein interaction
IGV	Integrative Genomics Viewer
γ-H2AX	Phosphorylated H2AX
DSBs	Double-strand breaks
