# Supplementary material for: Comprehensive Transcriptomic and m6A Epitranscriptomic Analysis Reveals Colchicine-Induced Kidney Toxicity via DNA Damage and Autophagy in HK2 Cells
Source: Toxins (Basel). 2025 Aug 14;17(8):408. doi: 10.3390/toxins17080408 (PMC12390427; doi:10.3390/toxins17080408)
Supplement: Supplementary file 1 [file toxins-17-00408-s001.zip › Table S1.pdf]

Table S1. The m6A modification levels of regulators in colchicine-treated HK2 cells.

Gene	Regulation	Chr	ChromStart	ChromEnd	log_2Fold Change	*p* Value	FDR
IGF2BP3	reader	chr7	23,351,777	23,352,376	0.31	6.38×10^{-4}	3.94×10^{-3}
ZC3H13	writer	chr13	46,585,574	46,626,069	−0.42	7.74×10^{-10}	1.58×10^{-8}
ZC3H13	writer	chr13	46,539,425	46,554,096	−0.15	2.59×10^{-11}	6.16×10^{-10}
METTL14	writer	chr4	119,606,546	119,612,725	−0.40	1.26×10^{-6}	1.54×10^{-5}
METTL14	writer	chr4	119,631,168	119,631,543	0.65	3.13×10^{-7}	4.26×10^{-6}
HNRNPC	reader	chr14	21,731,476	21,737,587	0.82	6.50×10^{-32}	5.31×10^{-30}
HNRNPC	reader	chr14	21,679,295	21,681,198	−0.39	2.43×10^{-116}	1.21×10^{-113}
CBLL1	writer	chr7	107,395,931	107,399,707	0.41	2.85×10^{-8}	4.71×10^{-7}
HNRNPA2B1	reader	chr7	26,237,039	26,240,366	−0.19	2.41×10^{-17}	9.48×10^{-16}
HNRNPA2B1	reader	chr7	26,231,746	26,236,939	−0.30	1.20×10^{-201}	1.63×10^{-198}
VIRMA	writer	chr8	95539,534	95,543,210	−0.25	3.94×10^{-8}	6.32×10^{-7}
VIRMA	writer	chr8	95,531,288	95,531,613	0.72	6.12×10^{-5}	5.08×10^{-4}
YTHDF1	reader	chr20	61,834,933	61,835,158	0.83	1.33×10^{-4}	1.01×10^{-3}
YTHDF1	reader	chr20	61,827,906	61,834,908	0.22	5.81×10^{-4}	3.64×10^{-3}
WTAP	writer	chr6	160,170,451	160,176,702	0.41	6.15×10^{-11}	1.40×10^{-9}
FMR1	reader	chrX	147,014,102	147,018,106	−0.33	3.11×10^{-4}	2.12×10^{-3}
FMR1	reader	chrX	147,024,714	147,030,279	−0.29	6.70×10^{-9}	1.22×10^{-7}
IGF2BP2	reader	chr3	185,407,368	185,538,819	−0.28	3.30×10^{-5}	2.96×10^{-4}

IGF2BP2	reader	chr3	185,404,930	185,404,955	−0.57	3.56×10^{-4}	2.39×10^{-3}
IGF2BP2	reader	chr3	185,393,203	185393662	−0.45	2.27×10^{-13}	6.58×10^{-12}
IGF2BP2	reader	chr3	185,362,942	185,367257	−0.15	1.14×10^{-5}	1.14×10^{-4}
IGF2BP2	reader	chr3	185,362,642	185,362,792	−0.28	1.17×10^{-3}	6.60×10^{-3}
ALKBH5	eraser	chr17	18,111,909	18,112,009	0.45	4.42×10^{-3}	2.00×10^{-2}
ALKBH5	eraser	chr17	18,112,984	18,113,134	0.67	6.26×10^{-3}	2.66×10^{-2}
IGF2BP1	reader	chr17	47,123,270	47,123,633	−0.38	2.75×10^{-4}	1.91×10^{-3}
RBM15	writer	chr1	110,882,494	110,884,519	0.27	1.39×10^{-5}	1.35×10^{-4}
RBM15	writer	chr1	110,884,544	11,088,8997	0.57	2.92×10^{-5}	2.65×10^{-4}
YTHDF3	reader	chr8	64,098,734	64,122,471	0.26	6.39×10^{-7}	8.19×10^{-6}
YTHDF2	reader	chr1	29,064,823	29,069,387	0.48	5.05×10^{-8}	7.94×10^{-7}
YTHDF2	reader	chr1	29,069,512	29,095,654	0.46	3.18×10^{-15}	1.08×10^{-13}
