# Supplementary material for: Comprehensive Transcriptomic and m6A Epitranscriptomic Analysis Reveals Colchicine-Induced Kidney Toxicity via DNA Damage and Autophagy in HK2 Cells
Source: Toxins (Basel). 2025 Aug 14;17(8):408. doi: 10.3390/toxins17080408 (PMC12390427; doi:10.3390/toxins17080408)
Supplement: Supplementary file 1 [file toxins-17-00408-s001.zip › Table S2.pdf]

Table S2. Sequence of siRNA-ZC3H13.

	siRNA sequence (5′-3′)	
	**sense strand**	**antisense strand**
si-NC	UUCUCCGAACGUGUCACGUTT	ACGUGACACGUUCGGAGAATT
si-ZC3H13	CAGCAUCAUUCUCCUAUAUTT	AUAUAGGAGAAUGAUGCUGTT
